# Identification of SARS‐CoV‐2 Omicron variant using spike gene target failure and genotyping assays, Gauteng, South Africa, 2021

**DOI:** 10.1002/jmv.27797

**Published:** 2022-05-08

**Authors:** Kathleen Subramoney, Nkhensani Mtileni, Avani Bharuthram, Ashlyn Davis, Beauty Kalenga, Mikateko Rikhotso, Mpho Maphahlele, Jennifer Giandhari, Yeshnee Naidoo, Sureshnee Pillay, Upasana Ramphal, Yajna Ramphal, Houriiyah Tegally, Eduan Wilkinson, Thabo Mohale, Arshad Ismail, Bonolo Mashishi, Nonhlanhla Mbenenge, Tulio de Oliveira, Zinhle Makatini, Burtram C. Fielding, Florette K. Treurnicht, Network for Genomics Surveillance in South Africa

**Affiliations:** ^1^ Department of Virology, National Health Laboratory Service Charlotte Maxeke Johannesburg Academic Hospital Johannesburg South Africa; ^2^ School of Pathology, Faculty of Health Sciences University of the Witwatersrand Johannesburg South Africa; ^3^ KwaZulu‐Natal Research Innovation and Sequencing Platform (KRISP), Nelson R Mandela School of Medicine University of KwaZulu‐Natal Durban South Africa; ^4^ Centre for Epidemic Response and Innovation (CERI), School of Data Science and Computational Thinking Stellenbosch University Stellenbosch South Africa; ^5^ Sequencing Core Facility National Institute for Communicable Diseases Johannesburg South Africa; ^6^ Department of Medical Biosciences, Molecular Biology and Virology Research Laboratory University of the Western Cape Bellville South Africa

**Keywords:** genotyping, Omicron BA.1, SARS‐CoV‐2, variants of concern

## Abstract

The circulation of Omicron BA.1 led to the rapid increase in severe acute respiratory syndrome coronavirus 2 (SARS‐CoV‐2) cases in South Africa in November 2021, which warranted the use of more rapid detection methods. We, therefore, assessed the ability to detect Omicron BA.1 using genotyping assays to identify specific mutations in SARS‐CoV‐2 positive samples, Gauteng province, South Africa. The TaqPath™ COVID‐19 real‐time polymerase chain reaction assay was performed on all samples selected to identify spike gene target failure (SGTF). SARS‐CoV‐2 genotyping assays were used for the detection of del69/70 and K417N mutation. Whole‐genome sequencing was performed on a subset of genotyped samples to confirm these findings. Of the positive samples received, 11.0% (175/1589) were randomly selected to assess if SGTF and genotyping assays, that detect del69/70 and K417N mutations, could identify Omicron BA.1. We identified SGTF in 98.9% (173/175) of samples, of which 88.0% (154/175) had both the del69/70 and K417N mutation. The genotyped samples (45.7%; 80/175) that were sequenced confirmed Omicron BA.1 (97.5%; 78/80). Our data show that genotyping for the detection of the del69/70 and K417N coupled with SGTF is efficient to exclude Alpha and Beta variants and rapidly detect Omicron BA.1. However, we still require assays for the detection of unique mutations that will allow for the differentiation between other Omicron sublineages. Therefore, the use of genotyping assays to detect new dominant or emerging lineages of SARS‐CoV‐2 will be beneficial in limited‐resource settings.

## 1 INTRODUCTION

The rapid evolution of severe acute respiratory syndrome coronavirus 2 (SARS‐CoV‐2) has led to the emergence of several variants of concern (VOC), which have been classified as variants with key characteristic features bearing significant epidemiological and clinical consequences.^1^ In 2020, the Wuhan lineage was replaced by B.1 and B.1.1 lineages, after which the Beta (B.1.351 lineage) VOC, Delta (B.1.617.2 lineage) VOC, and Omicron (B.1.1.529 lineage) VOC.^2^ In South Africa, the initial circulating Wuhan strain, in addition to several other SARS‐CoV‐2, was replaced by Beta, marking the first emergence of a VOC in the country. The Beta variant was first detected in South Africa in late 2020 and dominated from October 2020 to May 2021 during the second wave of infections; Delta dominated the third wave from June to November 2021; while the C.1.2 lineage (variant under monitoring) was detected at very low frequencies from July to December 2021.^3^, ^4^, ^5^, ^6^ In South Africa, recently emerged Omicron (B.1.1.529 lineage) was initially identified in a patient declared COVID‐19 positive on November 9, 2021, and since then Omicron has dominated the fourth wave.^7^ Since the initial detection of Omicron, the number of cases increased more rapidly compared to previous waves.^7^, ^8^

Omicron has over 30 mutations in the spike (*S*) protein, some of which overlap with the Alpha (del69/70, P681H), Beta (K417N, N501Y), and Delta (G142D and T478K) VOCs.^7^, ^9^, ^10^, ^11^ A number of these mutations, including the del69/70, are predicted or known to have an impact on immune escape or transmissibility.^11^ Since the discovery of Omicron (B.1.1.529 lineage), this VOC has been split into four sublineages namely BA.1, BA.1.1, BA.2, and BA.3. Omicron BA.1 isolates harbor the del69/70 in the S protein and were dominant from November 2021, Omicron BA.2 isolates do not have the del69/70 and started circulating in South Africa from December 2021, while the del69/70 is not present in Omicron BA.2 but is present in Omicron BA.3.^12^ The del69/70 affects the amplification of the S gene target during polymerase chain reaction (PCR) resulting in the S gene target failure (SGTF) detected by specific real‐time PCR assays.^13^, ^14^ The latter was found to be significant for the detection and reporting of Omicron.^13^, ^14^

Although next‐generation sequencing (NGS) remains the ideal tool for surveillance and detection of novel SARS‐CoV‐2 VOCs, many low‐ to middle‐income countries are unable to effectively implement this tool due to lack of resources, facilities, or expertise. Inconsistencies in testing and time delays in generating and releasing sequencing data were reported to hinder surveillance initiatives in African countries.^5^ Although still posing many challenges, the implementation of molecular diagnostic testing is far more achievable when coordinated efforts are made in comparison to the implementation of NGS.

In this study, we investigated the use of the Thermo Fisher Scientific TaqPath™ COVID‐19 assay and the SARS‐CoV‐2 genotyping assays to rapidly identify infections that occurred due to Omicron BA.1 sublineage, at the start of the fourth wave in South Africa, to highlight the usefulness and importance of molecular assays, especially in settings where NGS may not be readily available.

## 2 MATERIALS AND METHODS

### 2.1 Study population

The study cohort includes persons of all ages for whom upper respiratory tract samples were received for SARS‐CoV‐2 diagnosis, at the National Health Laboratory Service, Virology Laboratory, Charlotte Maxeke Johannesburg Academic Hospital the primary SARS‐CoV‐2 diagnostic testing facility in the City of Johannesburg Metropolitan district of Gauteng Province, from November 1 to 30, 2021. This includes samples collected from in‐patients, out‐patients, and community surveillance.

### 2.2 Study samples

The majority of respiratory samples received included nasal/nasopharyngeal and/or oropharyngeal swabs in viral or inactivation transport medium (Wuxi NEST Biotechnology), or dry swabs reconstituted in the laboratory in 1 ml phosphate‐buffered saline or viral transport medium.

### 2.3 Total nucleic acid extractions

Total nucleic acids (TNA) were extracted from 200 µl of respiratory specimens using the fully automated MagNA Pure 96 (MP96) extraction instrument coupled with the DNA and viral NA small volume TNA kit (Roche Diagnostics Mannheim), as per manufacturer's instructions. TNA extraction was also performed on the micro lab NIMBUS automated extraction system using the STARMag 96 × 4 Viral DNA/RNA 200 C Kit (Seegene Inc.).

### 2.4 SARS‐CoV‐2 diagnostics

Multiplex real‐time reverse‐transcription‐based‐PCR assays were performed on extracted TNA, according to the manufacturers' instructions. The assays performed included: a) the Allplex™ 2019‐nCoV Assay (Seegene Inc.) which targets the nucleocapsid (N), envelope (E), and RNA‐dependant RNA polymerase genes, was run on the CFX96 real‐time platform (Bio‐Rad Laboratories); b) the TaqPath™ COVID‐19 assay, which targets the N, open‐reading frame 1ab (ORF1ab) and spike (S) genes, was run on the QuantStudio™ 5 real‐time platform (Thermo Fisher Scientific); and c) the Biofire FilmArray RP2.1 assay that targets the S and membrane genes, was run on the BioFire Torch platform (BioFire Diagnostic).

### 2.5 Detection of del69/70 and the K417N using PCR‐based genotyping method

#### 2.5.1 Sample selection

Samples that tested positive for SARS‐CoV‐2, from the diagnostic SARS‐CoV‐2 PCR conducted from November 15 to 25, 2021, were randomly selected across collection date and site, regardless of the *C*_t_‐value. This period was selected since the detection rate of SARS‐CoV‐2 began to steadily increase.

#### 2.5.2 TNA extraction

TNA was extracted from the selected samples using the semi‐automated KingFisher Flex purification system (Thermo Fisher Scientific) with the MagMAX™ Viral/Pathogen II Nucleic Acid Extraction Kit (Thermo Fisher Scientific), as per the manufacturer's instructions. Samples that were initially tested on the CFX and BioFire platforms were retested using the TaqPath™ COVID‐19 assay to detect SGTF. The SGFT was defined as any sample for which the S gene did not amplify, but the N and/or ORF1ab genes amplified with *C*_t_ < 38.

The TaqMan SARS‐CoV‐2 singleplex single‐nucleotide polymorphism (SNP) genotyping assays (Thermo Fisher Scientific) were performed using the QuantStudio 5 real‐time platform (Thermo Fisher Scientific), to detect the del69/70 and K417N mutations associated with Omicron BA.1. These assays differentiate between wild type (H69V70 and K417) and mutant (del69/70 and 417N) in each sample.

SARS‐CoV‐2 PCR assay analysis was performed on the QuantStudio 5 design and analysis V2.5.1 software. SNPs were confirmed using both the allelic discrimination plots and amplification plots. Alleles that clustered along the *x*‐axis (allele 1, VIC‐labeled) represented the homozygous wild‐type genotype, while alleles that clustered along the *y*‐axis (allele 2, FAM‐labeled) represented homozygous mutant genotype, and alleles that clustered between the *x*‐axis and *y*‐axis represented heterozygous genotypes where both the mutant and wild‐type were present. Amplification curves were also analyzed to confirm the presence of the mutant and/or wild‐type.

### 2.6 Genotyping by SARS‐CoV‐2 genome sequencing

Samples with *C*_t_ < 31 were randomly selected across collection dates and sites on a weekly basis for SARS‐CoV‐2 whole‐genome sequencing. As part of the Network for Genomics Surveillance in South Africa, samples were submitted to the KwaZulu‐Natal Research Innovation and Sequencing Platform (KRISP), the National Institute for Communicable Diseases (NICD), or sequencing was performed in‐house. Samples were amplified using the ARCTIC V4 primers^15^ and libraries were prepared using the Nextera XT DNA Library Prep Kit (Illumina) which were sequenced on the MiSeq platform (Illumina) or COVIDSeq (Illumina) library kits were used with the NextSeq platform (Illumina).^11^, ^16^ Sequence reads were assembled using the Genome Detective SARS‐CoV‐2 online tool (https://www.genomedetective.com/app/typingtool/virus/) by KRISP, the Exatype pipeline (https://sars-cov-2.exatype.com/) by the NICD and the Galaxy SARS‐CoV‐2 pipeline (https://usegalaxy.eu/) for in‐house sequence reads. The consensus sequences were uploaded onto the GISAID (https://www.gisaid.org/) for curation (Table S1). Sequences were then downloaded from GISAID for further analysis using the Nextstrain (https://clades.nextstrain.org) online tool for the construction of the phylogenetic trees and the Pangolin lineage assigner (https://pangolin.cog-uk.io) was used to confirm the lineages.

### 2.7 Data analysis

As the del69/70 and K417N are present in other VOCs, apart from Omicron BA.1, genotyping by PCR was used to identify VOCs using the following algorithm for results interpretation: Alpha VOC if the del69/70 is present and K417N absent, Beta if del69/70 is absent and K417N is present, Delta if del69/70 and K417N are absent, and Omicron BA.1 if del69/70 and K417N are present.

The prevalence and trends of SARS‐CoV‐2 variants and their associated mutations were analyzed across different age groups (<1, 1–4, 5–17, 18–24, 25–44, 45–60, and >60), gender, period (day/month), and patient status, from November 15 to 25, 2021.

## 3 RESULTS

### 3.1 Participant demographics and SARS‐CoV‐2 diagnosis

**Figure 1:**
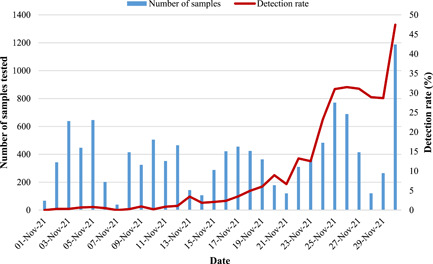
Detection rate of SARS‐CoV‐2 in Gauteng, South Africa, in November 2021. The bar graph represents the number of samples that were received at Virology, NHLS for SARS‐CoV‐2 diagnostics. The red line graph represents the detection rate (number of positive tests/number of samples received per day). NHLS, National Health Laboratory Service; SARS‐CoV‐2, severe acute respiratory syndrome coronavirus 2.

For the period November 1–30, 2021, a total of 11 549 diagnostic tests for SARS‐CoV‐2 were performed, of which 1589 (13.8%) samples tested positive. The number of samples received and tested declined from November 6th to 7th, 13th to 14th, 20th to 21st, and 27th to 28th, which was indicative of weekends (Figure 1). The detection rate of SARS‐CoV‐2 was below 6.7% from November 1st to 21st, 2021, and by the last week of November (22nd to 30th), the detection rate increased steadily up to 47.5% (Figure 1). From November 15 to 25, 32.6% (175/536) of positive samples were randomly selected to assess SGTF and genotyping by PCR. Among these, 57.7% (101/175) were females. Community surveillance samples represented 69.7% (122/175), in‐patient samples 9.1% (16/175), and out‐patients 13.1% (23/175) (Table 1). Among both males (43/71; 60.6%) and females (54/101; 53.5%), the majority of participants were 25–44 years of age (Table 1).

**Table 1:** Prevalence of S‐gene target failure (SGTF) among patients who tested positive for SARS‐CoV‐2 (*N* = 175).

Gender	Age (y)	SGTF present, *n*/*N *(%)			
		Community screening	In‐patient	Out‐patient	Unknown
Female (101/175; 57.7%)	<5 (*n* = 1)	1/70 (1.4)	–	–	–
	5–24 (*n* = 26)	18/70 (25.7)	3/13 (23.1)	2/14 (14.2)	2/3 (66.7)
	25–44 (*n* = 54)	39/70 (55.7)	6/13 (46.2)	9/14 (64.3)	‐
	45–60 (*n* = 16)	10/70 (14.3)	2/13 (15.4)	3/14 (21.4)	1/3 (33.3)
	>60 (*n* = 1)	1/70 (1.4)	–		–
	Unknown (*n* = 3)	1/70 (1.4)	2/13 (15.4)	–	–
Male (71/175; 40.6%)	<5 (*n* = 0)	–	–		–
	5–24 (*n* = 19)	14/49 (28.6)	1/3 (33.3)	1/9 (11.1)	3/9 (33.3)
	25–44 (*n* = 43)	30/49 (61.2)	1/3 (33.3)	6/9 (66.7)	5/9 (55.6)
	45–60 (*n* = 7)	4/49 (8.2)	–	2/9 (22.2)	1/9 (11.1)
	>60 (*n* = 2)	1/49 (2.0)	1/3 (33.3)	–	–
Unknown (3/175; 1.7%)	<5 (*n* = 0)	–	–	–	–
	5–24 (*n* = 1)	1/3 (33.3)	–	–	–
	25–44 (*n* = 2)	2/3 (66.7)	–	–	–
	45–60 (*n* = 0)	–	–	–	–
	>60 (*n* = 0)	–	–	–	–
Total^a^		122/175 (69.7)	16/175 (9.1)	23/175 (13.1)	12/175 (6.9)

Abbreviation: SARS‐CoV‐2, severe acute respiratory syndrome coronavirus 2.

^a^ No SGTF = 2.

**Figure 2:**
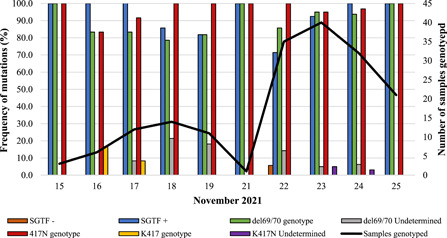
Frequency of SGTF, del69/70, and K417N mutations among SARS‐CoV‐2 positive samples collected from November 15 to 25, 2021. SGTF− represents the samples for which SGTF was absent. SGTF+ represents the samples with SGTF. From the genotyping PCR assay: The del69/70 genotype and del69/70 undetermined are samples with and without the deletion, respectively; and the K417, 417N, and K417N undetermined represent samples that had the wild‐type, mutation and neither at amino acid position 417, respectively. Genotyping was not performed on positive samples collected on November 20th. PCR, polymerase chain reaction;SARS‐CoV‐2, severe acute respiratory syndrome coronavirus 2; SGTF, spike gene target failure.

Of the 175 samples that were genotyped, 55% (97/175) were initially tested with the Allplex 2019‐nCoV and BioFire RP2.1, and only 2.1% (2/97) of these did not have SGTF (Table 1) when retested with the TaqPath™ COVID‐19 assay. The remaining 78 samples, which were initially tested with the TaqPath™ COVID‐19 assay, displayed SGTF. Overall 98.9% (173/175) of our samples exhibited SGTF (Figure 2).

### 3.2 Detection of del69/70 and K417N by PCR‐based genotyping

The del69/70 and K417N mutations, characteristic of the Omicron BA.1 sublineage, were observed in 88.0% (154/175) of samples (Figure 2), of which 87/154 (56.5%) were among SARS‐CoV‐2 positives initially diagnosed with the Allplex 2019‐nCoV and BioFire RP2.1 assays. All mutations were homogeneous (Figure S1). Among the samples that did not display SGTF (4/175; 2.3%), one did not have the del69/70 but the 417N mutation was present, and one had both the 417N and the del69/70 (both were confirmed by sequencing as BA.1) (Figure 2) whereas the remaining two samples had the wild‐type genotype at amino acid positions 69/70 and 417. We were unable to determine the presence of del69/70 for 8.0% (14/175) samples as a result of processing error when performing the genotyping assays as all samples had SGTF and confirmed N gene *C*_t_‐values of 15.4–34.8 from TaqPath COVID‐19 assay (Table S2). Only 28.6% (4/14) of samples were sequenced and were confirmed Omicron BA.1, of which 100% (4/4) had the del69/70 mutation, 50% (2/4) had the K417N mutation and the remaining two had a K417KN mixed mutation.

### 3.3 Confirmation of genotypes by sequencing

**Figure 3:**
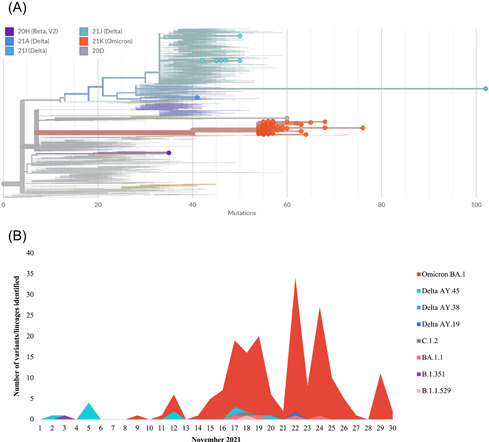
Phylogenetic representation of sequences from samples from Virology, NHLS Gauteng. Only sequence data from November 1st to 30th, 2021 from Virology, NHLS are represented in this figure. (A) Virology, NHLS sequence data are plotted against SARS‐CoV‐2 global data from the next clade (https://clades.nextstrain.org). The *X*‐axis represents the number of mutations present. The orange dots: Omicron (B.1.1.529/21K) and its sublineages including BA.1 (21K) and BA.1.1 (21K), turquoise dots: Delta sublineage AY.45 (21J), light blue dots: Delta sublineage AY.38 (21I), dark blue dots: Delta sublineage AY.19 (21A), purple dot: Beta (20H) and gray circle: C.1.2 (20D). Figure adapted from clades. nextstrain.org. (B) Representation of all Virology, NHLS sequence data was plotted for the period of November 1–30, 2021. From the 1st to 14th, the number of positive SARS‐CoV‐2 cases was few or completely lacking and therefore sequence data is very low or absent. NHLS, National Health Laboratory Service; SARS‐CoV‐2, severe acute respiratory syndrome coronavirus 2.

A total number of 242 samples were sequenced from November 1 to 30, 2021, of which 77.3% (187/242) were successful. Among successful sequences, Omicron BA.1 sublineage (170/187) was dominant, followed by Omicron BA.1.1 sublineage (4/187), B.1.1.529 sublineage (1/187), Delta (9/187), Beta (1/187), and C.1.2 (1/187) (Figure 3). Forty‐three percent (80/187) of samples genotyped by PCR were sequenced and the remaining 57.2% (107/187) were genotyped by sequencing.

Of the samples genotyped by PCR, sequencing confirmed that 97.5% (78/80) were Omicron BA.1 sublineage (21K clade), while the other two belonged to the B.1.1.529 lineage and Delta sublineage AY.19 (Figure 3). In our study population among the identified Omicron variants, we confirmed the characteristic genotype mutation K417N in 63.8% (51/80), 20.0% (16/80) were heterogenous at position 417 (K417KN) and 91.3% (73/80) del69/70 from our sequence data. For the remaining 13.8% (11/80) were wild‐type K417, 2.5% (2/80) had missing sequence data at position 417 whereas for positions 69/70, 2.5% (2/80) did not have the deletion, 5.0% (4/80) had missing sequence data, and 1.25% (1/80) had missing sequence data at amino acid position 69/70 but had A67ADGV and I68IM mutations. Thirty‐two additional mutations were observed in the S protein including A67V (91.3%; 73/80), T95I (92.5%; 74/80), G142D (91.3%; 73/80), del143‐145 (91.3%; 73/80), N211I (76.3%; 61/80), del212 (76.3%; 61/80), R214REPE (75%; 60/80), G339D (83.8%; 67/80), S371L (66.3%; 53/80), S373P (66.3%; 53/80), S375F (66.3%; 53/80), N440K (81.3%; 65/80), G446S (66.3%; 66/80), S477N (83.8%; 67/80), T478K (95%; 76/80), E484A (66.3%; 66/80), Q493R (66.3%; 66/80), G496S (81.3%; 65/80), Q498R (81.3%; 65/80), N501Y (81.3%; 65/80), Y505H (81.3%; 65/80), T547K (92.5%; 74/80), D614G (100%; 80/80), H655Y (100%; 80/80), N679K (96.3%; 77/80), P681H (97.5%; 78/80), N764K (91.3%; 73/80), D796Y (92.5%; 74/80), N856K (91.3%; 73/80), Q954H (93.8%; 75/80), N969K (95%; 76/80), and L981F (96.3%; 77/80).

One‐hundred and seven samples were genotyped by sequencing only, for which we did not have residual samples to perform the genotyping. The sequence data confirmed that the dominant variant circulating was Omicron BA.1 (90.6%; 92/107) which had similar mutation profiles to the sequencing results from the samples that were genotyped by PCR. The remaining 15 belonged to Delta sublineages AY.45 and AY.38 (8.4%; 9/107), Beta (1.0%; 1/107), Omicron BA.1.1 (3.7%; 4/107), and C.1.2 lineage (1.0%; 1/107) (Figure 3A). Delta sublineages were detected from November 2nd to 17th, the Beta was detected on November 3rd, Omicron BA.1.1 was detected from November 17th to 24th, and C.1.2 was detected on November 18th (Figure 3B).

## 4 DISCUSSION

A number of SARS‐CoV‐2 mutations within the receptor‐binding domain have been previously reported to play a role in neutralizing antibody escape. These mutations include K417N, G446S, E484A, Q493R, N440K, S371L, and S375F, and have been identified in Omicron, which dominated South Africa's fourth wave.^17^, ^18^ When compared to previously dominant VOCs, Omicron displays the greatest diversity, with over 30 mutations including the S protein mutations del69/70, T95I, G142D, del143‐145, K417N, T487K, N501Y, N655Y, N679K, and P681H,^7^, ^9^, ^10^ which are also observed in the Alpha, Beta, and Delta variants.^9^, ^11^, ^12^

Omicron was first observed among the samples received in our laboratory on November 9th, 2021. In November 2021, the number of positive SARS‐CoV‐2 cases doubled on a daily basis, with the majority of cases being attributed to Omicron BA.1.^8^ Our data showed that at the beginning of the fourth wave, males and females in the 25–44 year age group belonged to the highest percentage that tested positive of SARS‐CoV‐2, of which were predominantly infected with Omicron.

This study describes our ability to identify Omicron BA.1 using real‐time PCR and PCR‐based genotyping assays for a more rapid detection method compared to sequencing. The TaqPath™ COVID‐19 assay targets the S gene of the virus, which does not amplify in the presence of the deletion at amino acid position 69/70, resulting in SGTF as described by the World Health Organization.^14^ From the initially published sequences, it was evident that Omicron BA.1 contains the 69/70 deletion which is associated with SGTF.^7^, ^10^, ^11^ A recent South African study used SGTF as a proxy for Omicron BA.1 by selecting samples with SGTF and *C*_t_ ≤ 30 for the N and/or ORF1ab genes.^19^ However, in the latter study, the SGTF was only confirmed if the testing laboratories used the TaqPath™ COVID‐19 assay. In our study, the majority of samples that had SGTF, including *C*_t_ < 38 for N and ORF1ab genes, also had the del69/70 and were confirmed Omicron BA.1 with sequencing.

Of note is the shared del69/70 in Omicron BA.1 and Alpha. In both VOCs, this deletion results in SGTF when detecting SARS‐CoV‐2 using the TaqPath™ COVID‐19 assay. As a result, it has been suggested that the use of del69/70 and SGTF for identifying Omicron BA.1 is not possible since these characteristics will not uniquely identify Omicron BA.1.^13^ While the Alpha variant was sparsely identified at the beginning of South Africa's third wave, it was quickly out‐competed by the dominant Delta VOC. However, from November to December 2021, Alpha was still circulating in other regions globally, including the United Kingdom, Canada, United States, Germany, Belgium, France, India, Georgia, Israel, and Italy, (data accessed January 28, 2022, https://microreact.org/project/1WCmB1aqTRTkfCa3S1F9wT-global-sars-cov-2-2021-10-082021-12-29?dfc=lineage%26dfo=equals%26dfv=B.1.1.7%26cbc=lineage). To accurately distinguish between Alpha and Omicron BA.1, we combined del69/70 genotyping with K417N (not present in Alpha) genotyping. The K417N mutation is also present in Beta, but del69/70 is not, and therefore, SGTF will not be observed. This combination of genotypes/mutations facilitated the development of a genotyping assay that can distinguish between these VOCs, despite shared mutations. We could, therefore, conclude that combined use of the del69/70 and K417N genotyping assays could be useful for the detection of Omicron BA.1 in laboratories that do not use the TaqPath™ COVID‐19 assay.

For the samples that were genotyped but did not amplify for either the mutant or the wild type, we speculate that this may be due to poor sample quality or inappropriate storage conditions which affected downstream processing since the genotyping assay was performed on samples with *C*_t_ < 38. In addition, mutations observed within close proximity to the amino acid positions being scrutinized, such as A67ADGV and I68IM, may be present in the primer binding sites which may inadvertently have a negative impact on the genotyping assay for mutations at position 69/70. Genotyping data was not collected on November 20 since the number of samples that tested positive for SARS‐CoV‐2 was relatively low on the day and residual samples were not available for testing. However, these samples were selected for sequencing, from which we were able to confirm that the Omicron BA.1 was present.

The first limitation of this study was the lack of availability of suitable controls (wild‐type and mutant) at the time to assess the sensitivity of the genotyping assays to detect each genotype. We, therefore, utilized known positives as controls in addition to the commercially available controls in the case where commercial controls were unsuccessful. Second, the sample size was very small in comparison to the number of positive SARS‐CoV‐2 cases detected during the selected period. Therefore, this may have skewed the data distribution (age, gender, and patient status) of individuals for which samples had SGTF. Second, the short period we focused on covered Omicron BA.1; however, from December 2021, Omicron BA.1.1, BA.2, and BA.3 started circulating in South Africa.^12^ Omicron BA.2 does not have the del69/70 while the BA.1.1 and BA.3 do. Consequently, this implies that we will not be able to differentiate between Omicron BA.1, BA.1.1, and BA.3 as they share both the del69/70 and K417N mutations while Omicron BA.2 has the K417N which may not be easily distinguishable from Beta. Among our study sample, BA.3 was not identified, and overall only 1.0% (15/1579) of BA.3 was reported from South Africa, in November 2021.^12^ In a study from Japan, their genotyping assay targeted the G339D mutation which is present in all three Omicron sublineages and T547K mutation which easily differentiates Omicron BA.1 from BA.2 and BA.3.^20^ These mutations are also present among our study samples and their inclusion may substantially improve ones ability to differentiate between VOCs in countries, apart from South Africa. Another study described specific mutations to discriminate between BA.1.1, BA.2, and BA.3.^21^ BA.1.1 can be uniquely identified with the R346K mutation; BA.2 with the T19I, del24‐26, A27S, V213G, T376A, and R408S; and BA.3 with the del216, none of which were identified among samples from this study.^21^

## 5 CONCLUSION

Sequencing remains the gold standard for the detection of novel VOCs. However, in low to middle‐income countries, which may lack the expertise, facilities, or access to resources to perform NGS, the use of genotyping assays coupled with routine surveillance may assist with the detection of Omicron BA.1, even if there is cocirculation of the Beta, Delta, and/or Alpha VOCs. However additional assays are required to differentiate between Omcicron BA.1, BA.2, and BA.3 sublineages. We have demonstrated that the identification of SGTF and/or the del69/70 mutation together with the K417N mutation will provide a rapid, cost‐effective, and reliable method for the detection of the Omicron BA.1. However, we acknowledge the need for additional assays to target specific unique mutations that will differentiate one lineage from another based on the identification of unique mutations, without the use of sequencing.

## AUTHOR CONTRIBUTIONS

Florette K. Treurnicht and Kathleen Subramoney contributed to the concept, study design, and drafting of the manuscript. Ashlyn Davis, Nonhlanhla Mbenenge, Avani Bharuthram, Mikateko Rikhotso, Nkhensani Mtileni, Beauty Kalenga, Mpho Maphahlele, Kathleen Subramoney, Bonolo Mashishi, Nonhlanhla Mbenenge, Zinhle Makatini and Florette K Treurnicht contributed to diagnostic testing and data analysis. Kathleen Subramoney, Nkhensani Mtileni, and Beauty Kalenga contributed to the laboratory processing of the genotyping assay and data analysis. Jennifer Giandhari, Eduan Wilkinson, Yeshnee Naidoo, Houriiyah  Tegally, Sureshnee Pillay, Upasana Ramphal, Yajna Ramphal, Avani Bharuthram, Nkhensani Mtileni, Kathleen Subramoney, Tulio de Oliveira, Arshad Ismail, Thabo Mohale and Network for Genomics Surveillance in South Africa contributed to generating whole‐genome sequences, curation of metadata, and analysis. Florette K. Treurnicht, Burtram C. Fielding and Kathleen Subramoney were involved with the initial drafting of the manuscript, which was reviewed by all authors.

## CONFLICTS OF INTEREST

The authors declare no conflicts of interest.

## ETHICS STATEMENT

This study has received ethical clearance from the University of the Witwatersrand (Clearance Certificate Number M210119).

## Supporting information

Supporting information.

Supporting information.

## Data Availability

Sequence data can be accessed on GISAID (https://www.gisaid.org/) using the accession numbers provided in Table S1. Additional data (genotyping by polymerase chain reaction) has not been made available apart from the figures and tables included in this paper.
