# Supplementary material for: Identification of SARS‐CoV‐2 Omicron variant using spike gene target failure and genotyping assays, Gauteng, South Africa, 2021
Source: J Med Virol. 2022 May 8;94(8):3676–84. doi: 10.1002/jmv.27797 (PMC9088381; doi:10.1002/jmv.27797)
Supplement: Supplementary file 1 — Supporting information. [file JMV-94-3676-s001.docx]

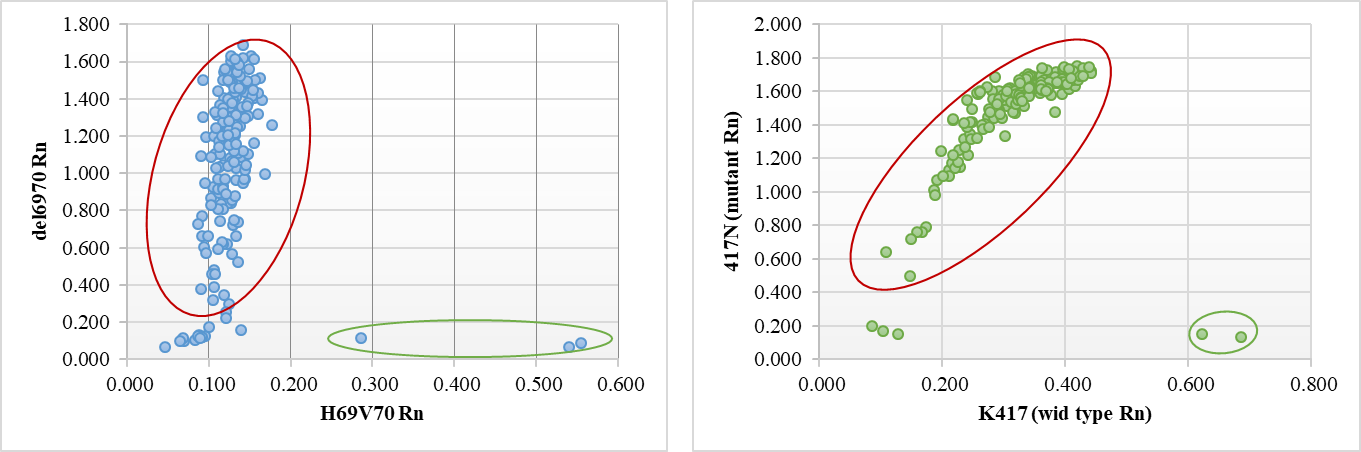


**Figure S1: Allelic discrimination plot of del69/70 (A) and K417N (B) mutations within the S gene showing florescence values (Rn).** Samples within the red oval (along the x-axis) represent homozygotes for either the deletion (A) or the 417N mutation (B). Samples within the green oval (along the y axis) represent those that do not have the deletion or have K417 wild type (homozygous wild type). Samples lying outside these areas represent a negative result (does not have the mutation or wild type).
