# Supplementary material for: Identification of SARS‐CoV‐2 Omicron variant using spike gene target failure and genotyping assays, Gauteng, South Africa, 2021
Source: J Med Virol. 2022 May 8;94(8):3676–84. doi: 10.1002/jmv.27797 (PMC9088381; doi:10.1002/jmv.27797)
Supplement: Supplementary file 2 — Supporting information. [file JMV-94-3676-s002.docx]

**Table S1: GISAID accession numbers for samples that were sequenced**

| EPI_ISL_7417383 | EPI_ISL_6699748 | EPI_ISL_7715352 | EPI_ISL_7715393 | EPI_ISL_7337485 |
| --- | --- | --- | --- | --- |
| EPI_ISL_7417285 | EPI_ISL_6699749 | EPI_ISL_7715353 | EPI_ISL_7747503 | EPI_ISL_7337486 |
| EPI_ISL_7417267 | EPI_ISL_6782071 | EPI_ISL_7715354 | EPI_ISL_7747504 | EPI_ISL_7337487 |
| EPI_ISL_7417129 | EPI_ISL_6699750 | EPI_ISL_7715355 | EPI_ISL_7852878 | EPI_ISL_7337488 |
| EPI_ISL_7417376 | EPI_ISL_6699751 | EPI_ISL_7715356 | EPI_ISL_7852884 | EPI_ISL_7337489 |
| EPI_ISL_7417384 | EPI_ISL_6782079 | EPI_ISL_7715357 | EPI_ISL_7852885 | EPI_ISL_7337490 |
| EPI_ISL_6795188 | EPI_ISL_6810484 | EPI_ISL_7715358 | EPI_ISL_7852886 | EPI_ISL_7337495 |
| EPI_ISL_6795189 | EPI_ISL_6699752 | EPI_ISL_7715359 | EPI_ISL_7852887 | EPI_ISL_7337496 |
| EPI_ISL_6795190 | EPI_ISL_6699753 | EPI_ISL_7715360 | EPI_ISL_7852888 | EPI_ISL_7337497 |
| EPI_ISL_6795191 | EPI_ISL_6699754 | EPI_ISL_7715361 | EPI_ISL_7852889 | EPI_ISL_7337498 |
| EPI_ISL_6795192 | EPI_ISL_6782080 | EPI_ISL_7715362 | EPI_ISL_7852890 | EPI_ISL_7337499 |
| EPI_ISL_6795193 | EPI_ISL_6782084 | EPI_ISL_7715363 | EPI_ISL_7852891 | EPI_ISL_7337500 |
| EPI_ISL_6795194 | EPI_ISL_6699755 | EPI_ISL_7715364 | EPI_ISL_9149845 | EPI_ISL_7337501 |
| EPI_ISL_6810481 | EPI_ISL_6699756 | EPI_ISL_7715365 | EPI_ISL_9149846 | EPI_ISL_7337502 |
| EPI_ISL_6699728 | EPI_ISL_6699757 | EPI_ISL_7715366 | EPI_ISL_9149847 | EPI_ISL_7337503 |
| EPI_ISL_6699729 | EPI_ISL_6810485 | EPI_ISL_7715367 | EPI_ISL_9149848 | EPI_ISL_7337504 |
| EPI_ISL_6810482 | EPI_ISL_6782090 | EPI_ISL_7715368 | EPI_ISL_9149849 | EPI_ISL_7337505 |
| EPI_ISL_6699730 | EPI_ISL_6699758 | EPI_ISL_7715369 | EPI_ISL_9149851 | EPI_ISL_7337506 |
| EPI_ISL_6699731 | EPI_ISL_6699759 | EPI_ISL_7715370 | EPI_ISL_9149852 | EPI_ISL_7337507 |
| EPI_ISL_6699732 | EPI_ISL_6699760 | EPI_ISL_7715371 | EPI_ISL_7337463 | EPI_ISL_7337508 |
| EPI_ISL_6782043 | EPI_ISL_6782091 | EPI_ISL_7715372 | EPI_ISL_7337464 | EPI_ISL_7337509 |
| EPI_ISL_6699733 | EPI_ISL_6699761 | EPI_ISL_7715373 | EPI_ISL_7337465 | EPI_ISL_7337510 |
| EPI_ISL_6699734 | EPI_ISL_6699762 | EPI_ISL_7715374 | EPI_ISL_7337466 | EPI_ISL_7337511 |
| EPI_ISL_6699735 | EPI_ISL_6699763 | EPI_ISL_7715375 | EPI_ISL_7337467 |  |
| EPI_ISL_6699736 | EPI_ISL_6699764 | EPI_ISL_7715376 | EPI_ISL_7337468 |  |
| EPI_ISL_6810483 | EPI_ISL_6699765 | EPI_ISL_7715377 | EPI_ISL_7337469 |  |
| EPI_ISL_6699737 | EPI_ISL_6699766 | EPI_ISL_7715378 | EPI_ISL_7337470 |  |
| EPI_ISL_6699738 | EPI_ISL_6810486 | EPI_ISL_7715379 | EPI_ISL_7337471 |  |
| EPI_ISL_6699739 | EPI_ISL_6699767 | EPI_ISL_7715380 | EPI_ISL_7337472 |  |
| EPI_ISL_6699740 | EPI_ISL_6810487 | EPI_ISL_7715381 | EPI_ISL_7337473 |  |
| EPI_ISL_6699741 | EPI_ISL_6699768 | EPI_ISL_7715382 | EPI_ISL_7337474 |  |
| EPI_ISL_6782048 | EPI_ISL_6699769 | EPI_ISL_7715383 | EPI_ISL_7337475 |  |
| EPI_ISL_6782055 | EPI_ISL_6782092 | EPI_ISL_7715384 | EPI_ISL_7337476 |  |
| EPI_ISL_6699742 | EPI_ISL_6699770 | EPI_ISL_7715385 | EPI_ISL_7337477 |  |
| EPI_ISL_6699743 | EPI_ISL_6699771 | EPI_ISL_7715386 | EPI_ISL_7337478 |  |
| EPI_ISL_6782056 | EPI_ISL_7715346 | EPI_ISL_7715387 | EPI_ISL_7337479 |  |
| EPI_ISL_6782066 | EPI_ISL_7715347 | EPI_ISL_7715388 | EPI_ISL_7337480 |  |
| EPI_ISL_6699744 | EPI_ISL_7715348 | EPI_ISL_7715389 | EPI_ISL_7337481 |  |
| EPI_ISL_6699745 | EPI_ISL_7715349 | EPI_ISL_7715390 | EPI_ISL_7337482 |  |
| EPI_ISL_6699746 | EPI_ISL_7715350 | EPI_ISL_7715391 | EPI_ISL_7337483 |  |
| EPI_ISL_6699747 | EPI_ISL_7715351 | EPI_ISL_7715392 | EPI_ISL_7337484 |  |

**Table S2:** **Comparison of cycle threshold (Ct) values of the TaqPath COVID-19 assay N gene target with those for the del69/70 and K417N genotypes.**

| **Sample ID** | **N gene (TaqPath COVID-19)*** | **del69/70 Call** | **del69/70 mutant ct value** | **K417N mutant ct value** | **K417N genotype** |
| --- | --- | --- | --- | --- | --- |
| Sample 33 | 34,8 | Undetermined | Undetermined | Homozygous M/M | 37,7 |
| Sample 116 | 20,5 | Undetermined | Undetermined | Homozygous M/M | 21,8 |
| Sample 117 | 22,8 | Undetermined | Undetermined | Homozygous M/M | 24,3 |
| Sample 118 | 32,3 | Undetermined | Undetermined | Homozygous M/M | 34,9 |
| Sample 119 | 31,2 | Undetermined | Undetermined | Homozygous M/M | 31,4 |
| Sample 127 | 15,4 | Undetermined | Undetermined | Homozygous M/M | 25,5 |
| Sample 128 | 21,8 | Undetermined | Undetermined | Homozygous M/M | 18,4 |
| Sample 129 | 35,3 | Undetermined | Undetermined | Homozygous M/M | 23,9 |
| Sample 131 | 30,3 | Undetermined | Undetermined | Homozygous M/M | 28,6 |
| Sample 145 | 33,9 | Undetermined | Undetermined | Homozygous M/M | 23,7 |
| Sample 153 | 26,7 | Undetermined | Undetermined | Homozygous M/M | 24,5 |
| Sample 162 | 28,5 | Undetermined | Undetermined | Homozygous M/M | 30,6 |
| Sample 163 | 28,5 | Undetermined | Undetermined | Homozygous M/M | 18,1 |
| Sample 8 | 19,2 | Homozygous M/M | 24,5 | Undetermined | Undetermined |
| Sample 11 | 18,6 | Homozygous M/M | 23,7 | Undetermined | Undetermined |
| Sample 15 | Undetermined | Undetermined | Undetermined | Undetermined | Undetermined |

*Samples for which the variant could not be accurately predicted, initial Ct values generated with the TaqPath COVID-19 assay (Thermofisher, ).
